# Inferring the Genetic Determinants of Fruit Colors in Tomato by Carotenoid Profiling

**DOI:** 10.3390/molecules22050764

**Published:** 2017-05-08

**Authors:** Hee Ju Yoo, Woo Jung Park, Gyu-Myung Lee, Chang-Sik Oh, Inhwa Yeam, Dong-Chan Won, Chang Kil Kim, Je Min Lee

**Affiliations:** 1 Department of Horticultural Science, Kyungpook National University, Daegu 41566, Korea; yhj901003@knu.ac.kr (H.J.Y.); codelkm@naver.com (G.-M.L.); ckkim@knu.ac.kr (C.K.K.); 2 Department of Marine Food Science and Technology, Gangneung-Wonju National University, Gangneung, Gangwon 25457, Korea; pwj0505@gwnu.ac.kr; 3 Department of Horticultural Biotechnology, College of Life Science, Kyung Hee University, Yongin, Gyeonggi 17104, Korea; co35@khu.ac.kr; 4 Department of Horticulture and Breeding, Andong National University, Andong, Gyeongbuk 36729, Korea; iyeam@andong.ac.kr; 5 Breeding Institute, Nongwoo Bio Co., Ltd., Yeoju, Gyeonggi 12655, Korea; dcwon7169@naver.com

**Keywords:** tomato, carotenoid, fruit color, HPLC, DNA marker, δ-carotene, prolycopene

## Abstract

Carotenoids are essential for plant and animal nutrition, and are important factors in the variation of pigmentation in fruits, leaves, and flowers. Tomato is a model crop for studying the biology and biotechnology of fleshy fruits, particularly for understanding carotenoid biosynthesis. In commercial tomato cultivars and germplasms, visual phenotyping of the colors of ripe fruits can be done easily. However, subsequent analysis of metabolic profiling is necessary for hypothesizing genetic factors prior to performing time-consuming genetic analysis. We used high performance liquid chromatography (HPLC), employing a C_30_ reverse-phase column, to efficiently resolve nine carotenoids and isomers of several carotenoids in yellow, orange, and red colored ripe tomatoes. High content of lycopene was detected in red tomatoes. The orange tomatoes contained three dominant carotenoids, namely δ-carotene, β-carotene, and prolycopene. The yellow tomatoes showed low levels of carotenoids compared to red or orange tomatoes. Based on the HPLC profiles, genes responsible for overproducing δ-carotene and prolycopene were described as *lycopene ε-cyclase* and *carotenoid isomerase*, respectively. Subsequent genetic analysis using DNA markers for segregating population and germplasms were conducted to confirm the hypothesis. This study establishes the usefulness of metabolic profiling for inferring the genetic determinants of fruit color.

## 1. Introduction

Tomato (*Solanum lycopersicum* L.) is a model crop for studying the biology of fleshy fruits, especially carotenoid biosynthesis [1]. In plants, carotenoids protect the cells from excessive radiation and render various colors, such as yellow, orange, and red to flowers, fruits, and vegetative organs [2]. The bright colors of flowers and fruits attract the pollinators and seed dispersers, and thereby, facilitate the propagation of plants. Carotenoids are precursors of plant hormones, such as abscisic acid (ABA) and strigolactones, as well as of various apocarotenoids [3]. Dietary carotenoids are essential for the health of humans as they are unable to synthesize carotenoids de novo [4]. Each carotenoid has its own function in promoting human health. For example, β-carotene, the precursor of vitamin A, is essential for the health of eye and prevents ocular consequences, cataract, and macular degeneration [5]. Similarly, lycopene protects against chronic diseases and decreases the risk of cancer and cardiovascular diseases [6]. Therefore, improving individual carotenoid levels is an important trait in the breeding of many crops.

**Scheme 1:**
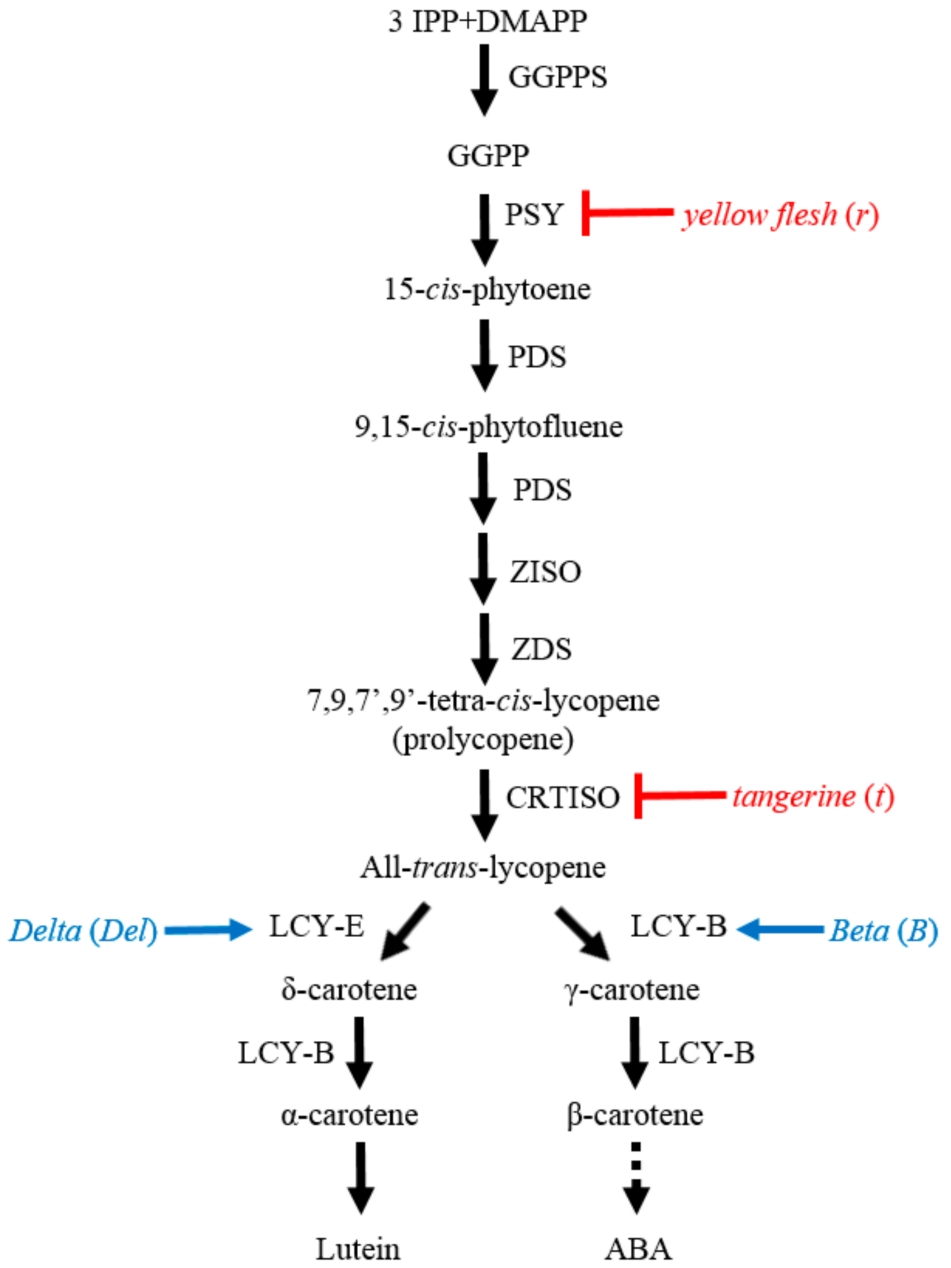
Carotenoid biosynthetic pathway in tomato. Mutants affecting the carotenoid biosynthesis are represented in italics. Blue color indicates positive regulation and red color indicates negative regulation of carotenogenesis. CRTISO, carotene isomerase; DMAPP, dimethylallyl diphosphate; GGPP, geranylgeranyl diphosphate; GGPPS, geranylgeranyl diphosphate synthase; IPP, isopentenyl diphosphate; LCY-B, lycopene β-cyclase; LCY-E, lycopene ε-cyclase; PDS, phytoene desaturase; PSY, phytoene synthase; ZDS, ζ-carotene desaturase; ZISO, ζ-carotene isomerase.

During ripening, the color of tomato fruit changes from green to red because of increased synthesis of carotenoids, especially lycopene. The carotenoid biosynthesis pathway was identified using genetic and biochemical approaches (Scheme 1) [7,8,9,10,11,12,13,14,15,16,17,18,19]. The two precursors of carotenoids, namely, isopentenyl diphosphate (IPP) and dimethylallyl diphosphate (DMAPP), are derived from the methylerythritol 4-phosphate (MEP) pathway. IPP and DMAPP are converted to geranylgeranyl diphosphate (GGPP) by geranylgeranyl diphosphate synthase (GGPPS). Subsequently, the carotenoid biosynthesis pathway is initiated by phytoene synthase (PSY), which condenses two GGPP molecules to form phytoene. Phytoene is desaturated by phytoene desaturase (PDS) to produce phytofluene. PDS, ζ-carotene isomerase (ZISO), and ζ-carotene desaturase (ZDS) catalyze the consecutive steps in the synthesis of *cis*-lycopene. Carotenoid isomerase (CRTISO) converts *cis*-lycopene to *trans*-lycopene by isomerization reactions at 7, 9 and 7′, 9′ *cis*-bonds. The *trans*-lycopene is cyclized through two routes involving lycopene ε-cyclase (LCY-E) and lycopene β-cyclase (LCY-B). The LCY-E and LCY-B catalyze the synthesis of δ-carotene and α-carotene, respectively, through one of the routes that branches at *trans*-lycopene and leads to the formation of lutein. The other route leads to the synthesis of γ-carotene and β-carotene by the catalysis of LCY-B and ultimately results in the formation of neoxanthin.

The accumulation of carotenoids is influenced by several factors, such as the intensity of radiation, CO_2_ concentration, temperature, photo-oxidative stress, and endogenous signals, and the biosynthetic genes are spatio-temporally regulated at the transcriptional level [20,21,22,23,24,25,26,27,28,29,30,31]. Several mutants have been used to study the carotenoid metabolism in tomato [2,17]. The *yellow flesh* (*r*) mutant (with loss of *PSY1* function) has yellow ripe fruits because of the lack of carotenoid synthesis during ripening [9]. The *tangerine* (*t*) mutant (with loss of *CRTISO* function) rarely produces *trans*-lycopene [12]. The *Delta* (*Del*, overexpressor of *LCY-E*) and *Beta* (*B*, overexpressor of *LCY-B*) mutants synthesize high amounts of δ-carotene and β-carotene, respectively, at the expense of *trans*-lycopene [15,16]. The *t*, *Del*, and *B* mutants show orange ripe fruits because of differences in the carotenoid composition compared to that in the red tomatoes.

The tomato mutants described above were identified by positional cloning and transgenic approaches, which are time-consuming. Several such mutants have been developed [32,33] and germplasms have been collected (for example, see the website of Tomato Genetics Resource Center, http://tgrc.ucdavis.edu/). It is necessary to classify the newly developed resources along with the previously identified resources. The classical phenotyping might not be able to resolve the genetic variations that show similar fruit colors, and time-consuming genetic analysis is impractical. However, prior to the genetic analysis, metabolite analysis can easily and quickly provide information for investigations on the genes involved in carotenoid accumulation in tomato fruits [34]. Direct metabolite analysis approach was developed and used to characterize mutants accumulating high or low amounts of metabolites [35]. Changes in metabolite contents suggest altered enzyme activity and gene expression [36]. Thus, analysis of carotenoid compounds in tomato mutants and germplasms showing different fruit colors can provide important insights into the genes associated with the color. Various methods, such as thin layer chromatography (TLC), liquid chromatography (LC), gas chromatography (GC), mass spectrometry (MS), and nuclear magnetic resonance (NMR), have been used for metabolite analysis [37]. Among these, HPLC combined with UV-Vis detection is especially a well-known method for identification and quantification of carotenoids because of its speed, high resolution, and relatively easy handling [38,39,40]. Carotenoids are usually separated by reverse-phase HPLC using a C_30_ stationary phase column and are identified by determining the absorption spectrum with a photodiode array detector (PDA) because different carotenoids have unique structures and different absorption maxima [39,41]. In this study, we investigated the simultaneous use of metabolite profiling and genetic analysis for identifying the genes regulating the fruit color and carotenoid accumulation in tomato.

## 2. Results and Discussion

### 2.1. Separation and Identification of Carotenoid Compounds in Red Tomato Fruits Using HPLC

**Scheme 2:**
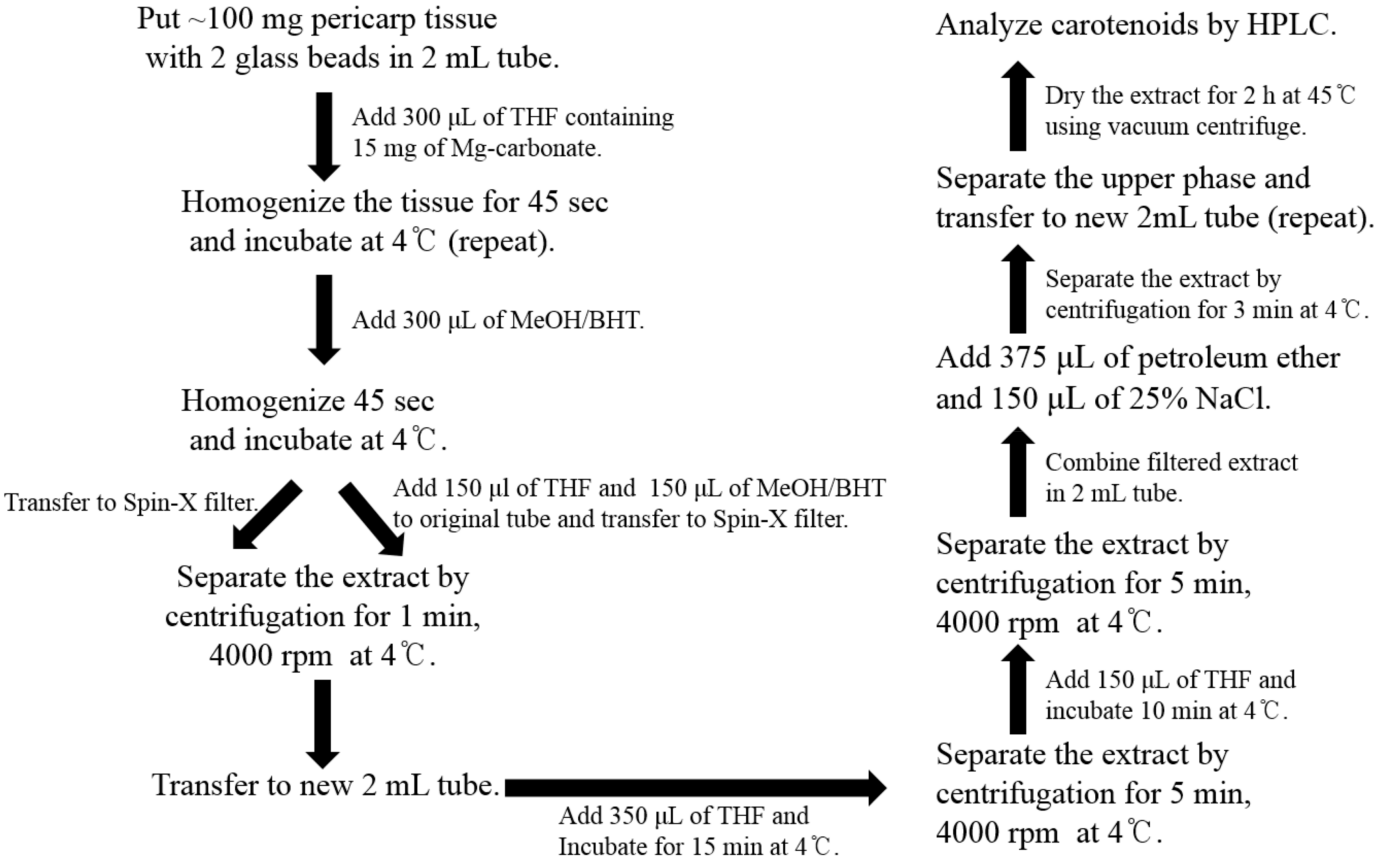
Procedure for carotenoid extraction from the tomato pericarp. THF, tetrahydrofuran; MeOH, methanol; BHT, butylated hydroxytoluene; NaCl, sodium chloride.

**Figure 1:**
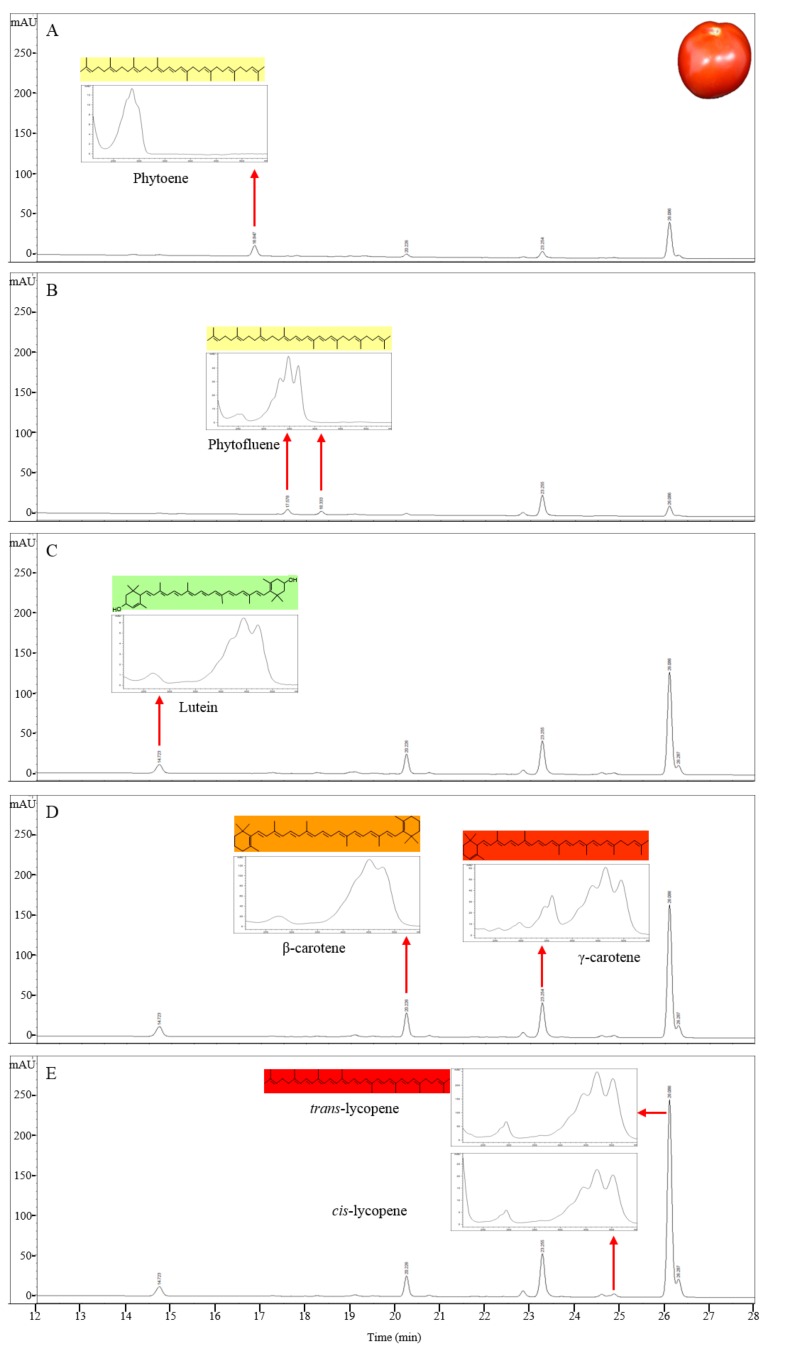
HPLC profile of carotenoids as determined by their absorption between 286 and 471 nm in red tomato (LA3475). The carotenoid compounds were detected by their absorption at five wavelengths and were identified by their absorption maxima and spectrum. (**A**) 286 nm; (**B**) 348 nm; (**C**) 434 nm; (**D**) 450 nm; (**E**) 471 nm.

To separate and identify the carotenoid compounds in red tomatoes, carotenoids were extracted from the pericarp of *S. lycopersicum* ‘LA3475’ (Scheme 2) and then analyzed by HPLC using a C_30_ reverse-phase column and PDA (Figure 1). All the cultivars in this study were of *S. lycopersicum* and only the cultivar names are used hereafter. The retention time and absorption spectra of the carotenoid compounds in the ripe tomatoes of LA3475 were determined by HPLC and used as reference profiles with which the carotenoid profiles of orange and yellow tomatoes were compared. In a run time of 36 min, carotenoids were detected over a wavelength range from 286 to 471 nm. Seven major carotenoids were identified based on the retention times and absorption spectra identified previously [39,41], with each carotenoid showing a unique absorption spectrum (Table 1 and Figure 1). Phytoene, phytofluene, and lutein were identified based on their absorption at 286 nm, 348 nm, and 434 nm, respectively. β-Carotene and γ-carotene were identified on the basis of their absorption at 450 nm, and *cis*- and *trans*-lycopene were identified on the basis of their absorption at 471 nm. The major component in the red tomato was *trans*-lycopene, as is already known, and it was detected last at a retention time of 26.4 min. The shape of absorption spectrum, showing the presence of carotenoids, was clear in all the compounds.

### 2.2. Analysis of Carotenoid Profiles in Tomatoes Showing Various Colors of Ripe Fruits 

**Figure 2:**
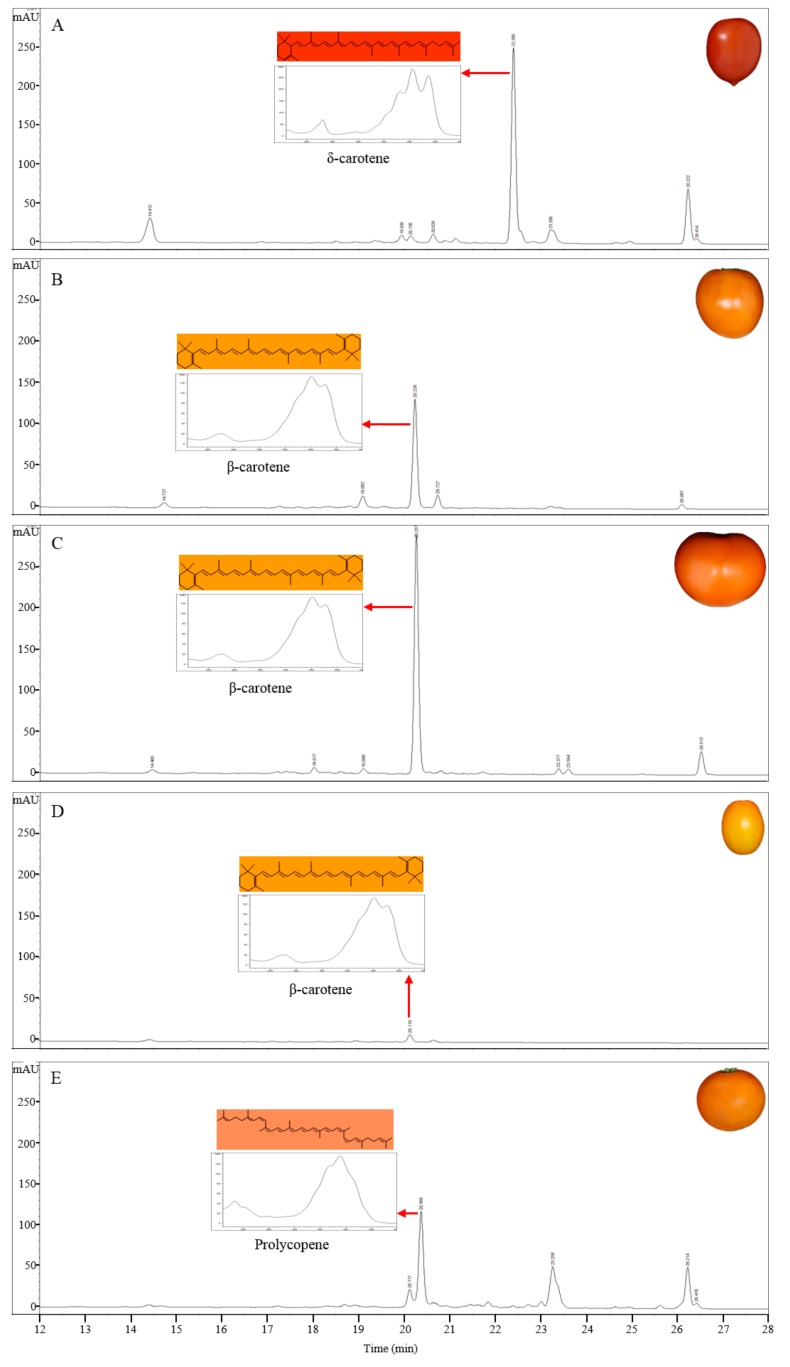
HPLC profiles of carotenoids in tomato germplasms as determined by their absorption at 450 nm. (**A**) LA4099; (**B**) LA3311; (**C**) LA4255; (**D**) Sugar Yellow; (**E**) LA3533. Eight other lines (LA0030, LA0351, LA3002, LA3128, LA3183, LA3682, Cispene, and Gold Minichal) accumulating prolycopene showed similar HPLC profiles.

The carotenoid compounds were extracted from ripe tomato fruits of different colors and were analyzed using HPLC (Figure 2). The composition of carotenoids in the tomato fruits showing yellow and orange colors was clearly different from that in LA3475 tomato with red colored fruits. The major carotenoid peak in LA4099, which has dark orange colored fruits, was detected at 450 nm at a retention time of 22.3 min; this peak was not detected in LA3475 (Figure 2A). The component detected at 22.3 min was identified as δ-carotene based on the absorption maxima and spectrum (Table 1). Compared to the peaks in LA3475, a reduced peak of *trans*-lycopene at a retention time of 26.4 min and an increased peak area of lutein at a retention time of 14.4 min were observed. δ-Carotene is synthesized by LCY-E at the expense of *trans*-lycopene and is a precursor of lutein. Therefore, we assume that the higher accumulation of δ-carotene and lutein was due to the higher expression or activity of *LCY-E*.

The orange colored fruits of LA3311 and LA4255 showed higher peak of β-carotene coupled with reduced peak area of *trans*-lycopene compared to those in LA3475 (Figure 2B,C). β-Carotene is synthesized by LCY-B at the expense of lycopene. Therefore, being a δ-carotene over-producing line, high expression or activity of LCY-B is a major determinant in the high accumulation of β-carotene.

The HPLC profile in Sugar Yellow tomato with yellow-colored fruits was different from that of the other tomato lines. The β-carotene content was much lower than that of other lines and the level of other carotenoids were also very low. Furthermore, lycopene was not detected (Figure 2D). These results indicate that Sugar Yellow is mutated in a gene upstream of carotenoid or isoprenoid biosynthesis such as *yellow flesh* (*r*) [11] or *apricot* (*at*) [42], showing lack of carotenoids in ripe tomato fruits. In the *at* mutant, the flower color showed deficiency of carotenoids unlike in the *r* mutant. The flower color in Sugar Yellow was same as that in the wild type (data not shown) suggesting that the *at* mutation may not affect the fruit color variation in Sugar Yellow. The ripening mutants normally showed lack of carotenoid in addition to the ripening phenotypes, such as higher fruit firmness. Sugar Yellow showed normal ripening patterns as in the wild type.

LA0030, LA3183, LA3533, LA3682, Gold Minichal, and Cispene with orange ripe fruit color contain lower amounts of β-carotene and lycopene compared to that in LA3475. HPLC analysis of these lines showed major absorption peaks at 450 nm at a retention time of 20.6 min, which were identified to be prolycopene based on the absorption maxima and spectrum (Figure 2E). Prolycopene (7,9,7′,9′-tetra-*cis*-lycopene), showed different absorption spectrum and retention time compared to *cis*-lycopene detected in red tomato. Thus, prolycopene is a representative carotenoid only present in these lines. Subsequently, these lines were assumed to be regulated by the mutation in *CRTISO* [13], which is responsible for *cis*- to *trans*-lycopene isomerization. Therefore, interrupting the synthesis of *trans*-lycopene may lead to the accumulation of its precursor, prolycopene, and the appearance of orange color.

### 2.3. Association Analysis between HPLC Profiles and Genotype Screening for Carotenoid Accumulation

**Figure 3:**
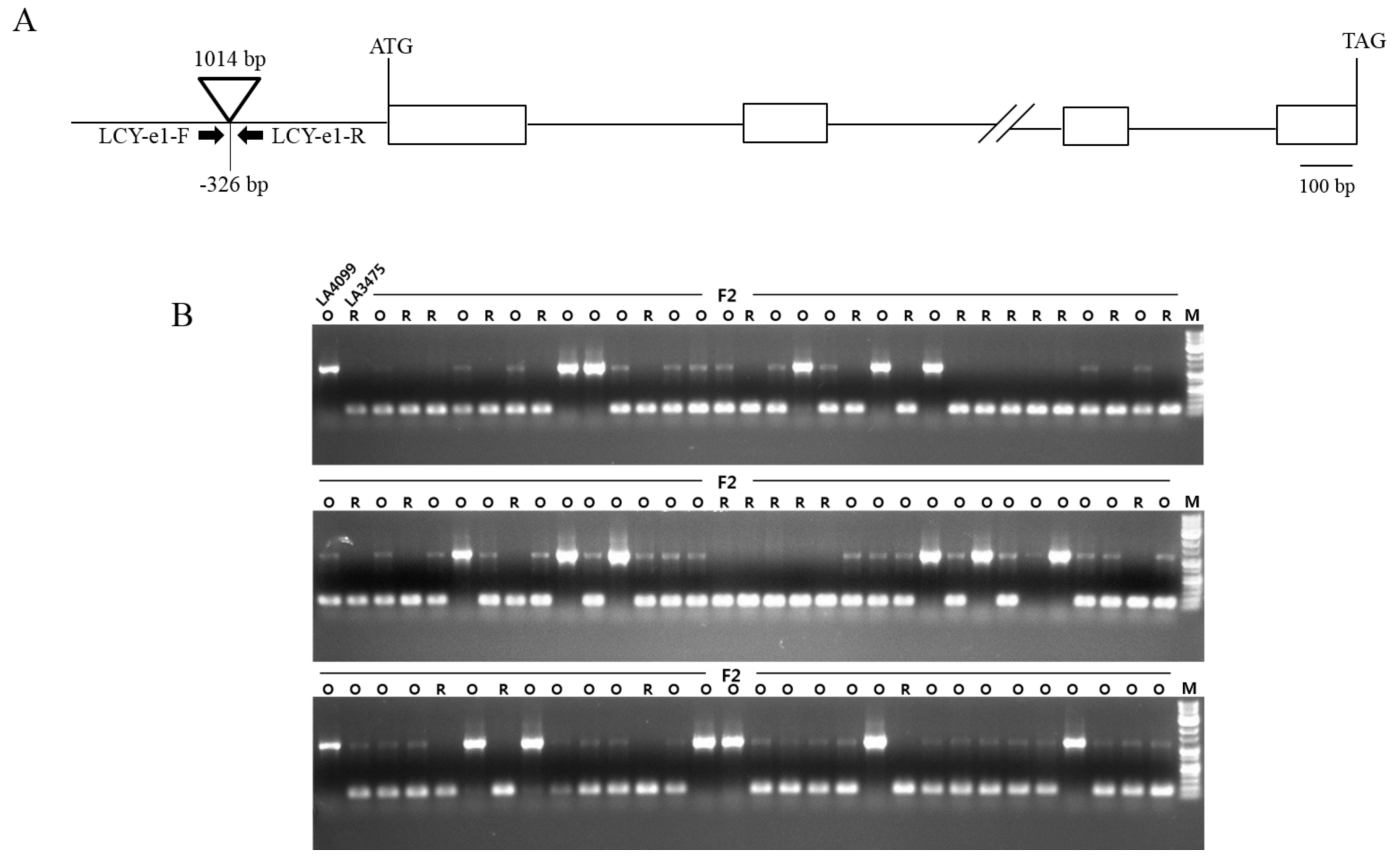
The variation in fruit color in LA4099 co-segregated with *LCY-E* in F_2_ population derived from a cross between LA3475 and LA4099. (**A**) Gene structure of *LCY-E* and its genetic variation in LA4099. A DNA fragment of 1014 bp was inserted at the promoter region of *LCY-E* in LA4099; (**B**) Co-segregation analysis between *LCY-E* and the dark orange fruit colors in F_2_ population of LA3475 × LA4099. The insertion at *LCY-E* was distinguished by a DNA marker (LCY-e1). The marker co-segregated with the fruit colors in 94 F_2_ progenies derived from a cross of LA3475 and LA4099. O and R represent the orange and red fruit colors, respectively, in the parental lines and F_2_ population. M indicates 1 kb DNA ladder.

The dark orange colored fruit in LA4099 contained a high amount of δ-carotene and a low amount of *trans*-lycopene compared to the amounts of these present in the red tomato fruit of LA3475 (Figure 2A). Based on the carotenoid profile and subsequent hypothesis-driven approach, *LCY-E* might be supposed to be mutated or up-regulated in LA4099. The changes in gene expression are often due to genetic variations in the promoter regions. Thus, promoter regions of *LCY-E* in LA4099 and LA3475 were sequenced and compared to identify the genetic variation. In LA4099, 1,014 bp nucleotides were inserted in the promoter region at −326 bp position from the start codon (Figure 3A). To identify this variation in LA4099, INDEL marker (LCY-e1) of *LCY-E* was developed (Table 2). To verify whether the fruit colors were genetically determined by the variation in *LCY-E*, LA3475 and LA4099 were crossed, and genetic and phenotypic analyses were conducted in their F_2_ population (Figure 3B). The F_1_ fruits of LA3475 × LA4099 showed orange color as in LA4099, and the F_2_ progenies segregated into those with orange (66 individuals) and red (28 individuals) colored ripe fruits at a ratio of 3:1 (χ^2^ = 1.15, *p*-value = 0.2838). The genotypes of *LCY-E* in the F_2_ population were analyzed using the INDEL marker. We could identify 19 homozygous individuals of the LA4099 type (orange fruits), 47 heterozygous individuals (orange fruits), 28 homozygous individuals of the LA3475 type (red fruits), as per the expected genotypic ratio of 1:2:1 (χ^2^ = 1.72, *p*-value = 0.4244). The genotypic data co-segregated with the phenotypic data, suggesting that *LCY-E* is the genetic determinant of the dark orange fruit color in the δ-carotene overproducer, LA4099.

The δ-carotene over accumulating tomato, with high activity of *LCY-E*, is a candidate source for increased lutein. In a previous study, transgenic tomato over-expressing *LCY-E* showed higher accumulation of δ-carotene. This transgenic line was crossed with the *Beta* mutant (overexpressing *LCY-B*) and the resulting hybrid line was shown to accumulate high amounts of lutein because of the over-expression of *LCY-E* and *LCY-B* [43]. Lutein, a macular pigment in addition to zeaxanthin, is a functional nutrient. In addition, lutein plays a role in the protection of eye by absorbing the blue light, which causes light-induced injury in the photoreceptor cells [44]. Therefore, δ-carotene-enriched line can be a useful source for improving the lutein content in tomato fruit.

**Figure 4:**
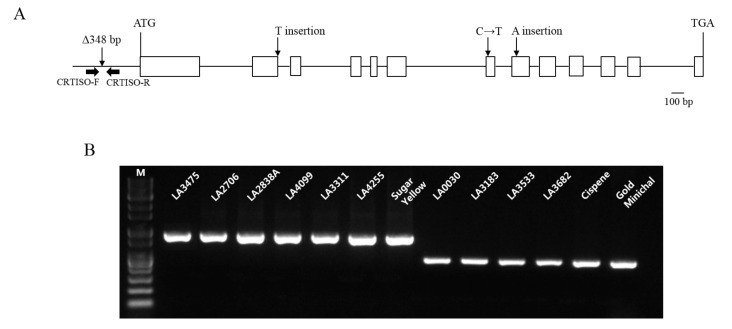
Gene structure of *CRTISO* and genotypic analysis of orange tomatoes enriched in prolycopene using the INDEL maker of *CRTISO*. (**A**) *CRTISO* mutation at the promoter region was previously identified [12] and three new mutations were identified in the coding region in this study. Two nonsense mutations were caused by one nucleotide insertion in the 2nd and 8th exons, respectively, and a missense mutation (C to T) was found in the 7th exon; (**B**) Deletion at the promoter region of *CRTISO* was distinguished by DNA marker (CRTISO) in prolycopene over-accumulated lines. Other tomatoes showing various colors, such as red (LA3475, LA2706, LA2838A), orange (LA4099, LA3311, LA4255), and yellow (Sugar Yellow) showed no deletion at the promoter region of *CRTISO*. M indicates 1 kb DNA ladder.

Nine tomato lines (LA0030, LA0351, LA3002, LA3128, LA3183, LA3533, LA3682, Cispene, and Gold Minichal) showing orange fruit color accumulated increased amounts of prolycopene and reduced amounts of *trans*-lycopene compared to LA3475 (Figure 2E). Because the candidate gene affecting this variation was inferred to be *CRTISO*, the variations in *CRTISO* in these lines were investigated. In a previous study, the *CRTISO* mutant, *tangerine*, was found to be enriched with prolycopene and had deletions of 282 bp and 348 bp in the 1st exon and 1st intron and promoter regions, respectively [12]. Among the two reported variations, the deletion of 348 bp at the promoter region was discovered in six lines (LA0030, LA3183, LA3533, LA3682, Cispene, and Gold Minichal) by the INDEL marker of *CRTISO* whereas three lines (LA0351, LA3002, and LA3128) did not contain the deletion (Figure 4, Table 2). Thus, the genomic DNAs of *CRTISO* in these lines were sequenced using five primers (Table 2) and compared with that of the wild type, LA3475. Nonsense mutations caused by the insertion of thymine in the 2nd exon (LA3002) and adenine in the 8th exon (LA3128) were discovered. A missense mutation was found to convert cytosine to thymine in the 7th exon in LA0351. In addition to the previously identified *tangerine* allele, these three alleles would be valuable for improving the prolycopene content in tomato breeding.

Prolycopene is one of the *cis*-lycopene isomers mainly present in the human blood and tissues although *trans*-lycopene is the principal form of lycopene in red tomato [45]. Humans efficiently absorb more *cis*-lycopene isomer from the *t* mutant tomato sauce compared to equal amounts of *trans*-lycopene from red tomato sauce in their total serum [46]. Thus, *CRTISO* mutants would be useful resources for improving the bioavailability of lycopene [47].

The red color of tomato is because of the higher accumulation of *trans*-lycopene, whereas carotenes and xanthophylls impart orange and yellow colors, respectively. Thus, it is difficult to distinguish the genetic variation visually because most of the tomato mutants with defect in the carotenoid biosynthesis genes show orange and yellow colored ripe fruits. In this study, we identified distinct carotenoid profiles of tomatoes showing diverse fruit colors and inferred the candidate genes for each variation in addition to the genetic analysis. Visual phenotypes were often connected with dramatic metabolic changes. Thus, metabolic profiling can be a highly efficient method to infer the genetic determinants regulating the metabolite synthesis or accumulation before conducting the time-consuming genetic analysis.

## 3. Materials and Methods 

### 3.1. Plant Materials

Thirteen tomato germplasms and three commercial cultivars (three red tomatoes, twelve orange tomatoes, and one yellow tomato) were used for the profiling of carotenoids. The tomato plants were grown in a greenhouse with 16 h light/8 h dark period at temperatures ranging from 19 to 28 °C, 50–70% relative humidity. After fruit setting, 2 g of water soluble NPK fertilizer (17 − 8 − 26 + 2 MgO with micro nutrient, Poly-Feed™ Drip, Haifa Chemicals Ltd., Matam-Haifa, Israel) per each plant were supplied every week. Three ripe fruits (ten days post-breaker stage) per each line were harvested for evaluation of fruit colors and extraction of carotenoids from the pericarp. The fruit colors were evaluated by three persons. The harvested pericarps were immediately frozen in liquid nitrogen and stored at −80 °C before the extraction of carotenoids.

### 3.2. Carotenoid Extraction

To prevent the degradation and oxidation of carotenoids, all the experiments were conducted under limited light conditions. For carotenoid extraction, the method of extraction described by Vrebalov et al. [25] was used with some modifications and a schematic diagram for the same is presented in Scheme 2. All the solvents used were of HPLC grade. Frozen tomatoes were ground and about 100 mg of the tissue powder was placed in a 2 mL tube with two glass beads (6 mm). The frozen tissues were homogenized with 15 mg of magnesium carbonate and 300 μL of tetrahydrofuran (THF) for 60 s using FastPrep-24™ instrument (MP Biomedicals, Santa Ana, CA, USA). The extracts were homogenized again after adding 300 μL of methanol (MeOH) containing 5% butylated hydroxyl-toluene (BHT). The homogenate was transferred to Spin-X centrifuge filter tube (0.45-mm nylon filter, Corning Incorporated, Corning, NY, USA) and centrifuged for 1 min at 4000 rpm at 4 °C. The original 2 mL tube was kept on ice and 150 μL of THF and 150 μL of MeOH, containing 5% BHT, were added to the tube. The tissue debris in the original tube was transferred using a cut tip to Spin-X centrifuge filter tube and then centrifuged for 1 min at 4000 rpm and 4 °C. The filtered extract was transferred to a new 2 mL tube. For the complete extraction of carotenoid, 350 μL of THF was added in Spin-X centrifuge filter tube, incubated on ice for 15 min, and then centrifuged at 4000 rpm for 5 min at 4 °C. This step was repeated. The filtered extract was combined with the previous extract in a 2 mL tube. To separate the carotenoid/nonpolar phase from the aqueous phase, 375 μL of petroleum ether and 150 μL of 25% sodium chloride were added into the filtered extract, vortexed vigorously, and centrifuged for 3 min. The upper phase was separated from the lower phase or inter-phase into a new 2 mL tube. For re-extraction, 500 μL of petroleum ether was added and the upper phase was separated as described for the previous step. The petroleum ether extract was dried using MICRO-CENVAC machine (NB-503CIR, N-BIOTEK, Bucheon, Korea) for 2 h at 45 °C. If HPLC was not performed immediately, the carotenoid extract was stored at −80 °C.

### 3.3. Carotenoid Analysis Using HPLC

The carotenoid extracts were suspended in 250 μL of methyl *t*-butyl ether and 242 μL of MeOH. The carotenoid suspension was filtered through a 0.45-μm syringe filter (SmartPor^®^-II NYLON Syringe filter with 13 mm, 0.45 µm, Woongki Science Co., Ltd., Seoul, Korea) and put in an HPLC vial. The carotenoid analysis was performed using a 1260 Infinity series HPLC instrument (Agilent, Santa Clara, CA, USA) and the Chemstation software. The injection volume was 25 μL and the flow rate was 1 mL min^−1^. The column temperature was maintained at 25 °C for all the analyses. The carotenoids were separated under a polar to nonpolar gradient using mobile phases consisting of 100% methanol with 0.1% ammonium acetate (A) and methyl t-butyl ether (B) through a guard column (YMC Guard Cartridge, YMC Co., Ltd., Kyoto, Japan) and C_30_ stationary phase (YMC Carotenoid S-5 μm, 250 × 4.6 mm, YMC Co., Ltd., Kyoto, Japan). A gradient elution was performed with 100% of A for 6 min, which was then gradated to 4% of A and 96% of B over 20 min. After 26 min, A was increased to 100% until 36 min. The elution of carotenoids was observed on an online Diode Array Detector (PDA) at five wavelengths (286 nm, 348 nm, 434 nm, 450 nm, and 471 nm). The carotenoids were identified based on their absorption maxima and spectrum [39,41].

### 3.4. DNA Extraction

The genomic DNA of tomato was extracted using a modified cetyltrimethylammonium bromide (CTAB) method [48]. The young leaf tissue was homogenized in FastPrep-24™ instrument (MP Biomedicals, Santa Ana, CA, USA) with two glass beads (6 mm), 600 μL of CTAB buffer (2% CTAB, 1.42 M NaCl, 20 mM ethylenediaminetetraacetic acid (EDTA), 100 mM Tris-Cl (pH 8.0)), 0.5% β-mercaptoethanol, and 1 mg of l-ascorbic acid in a 2 mL tube. The extract was incubated at 65 °C for 15 min. Thereafter, 600 μL of chloroform:isoamyl alcohol (24:1 *v*/*v*) was added and mixed well. The extract was centrifuged at 13,000 rpm for 15 min at 4 °C. The upper phase was transferred to a new tube and 500 μL of isopropanol was added and then the mixture was centrifuged at 13,000 rpm for 15 min at 4 °C. The supernatant was removed and 70% ethanol was added to the pellet and then centrifuged at 13,000 rpm for 5 min at 4 °C. Ethanol was removed and the extract was centrifuged for 1 min. The remaining ethanol was removed and 30 μL of ddH_2_O containing 1% RNase was added to the pellet.

### 3.5. Sequence Analysis and DNA Marker Analysis

Polymerase chain reaction (PCR) was conducted using *e-Taq* DNA polymerase (SolGent, Daejeon, Korea) in a T-100 Thermal Cycler (BIO-RAD, Hercules, CA, USA), with 100 ng of each DNA template and 10 pmol of gene specific primers for *LCY-E* (Solyc12g008980) and *CRTISO* (Solyc10g081650) (Table 2). The PCR conditions were 95 °C for 3 min, followed by 35 cycles of 95 °C for 1 min, 56 °C for 1 min, and 72 °C for 2 min. The amplified fragments were cloned into T-blunt vector (T-Blunt™ PCR Cloning Kit, SolGent, Daejeon, Korea), as per the manufacturer’s protocol, and transformed into *E. coli* (DH5α). The plasmid DNA was isolated by a plasmid prep kit (HiGene™ Plasmid Mini Prep Kit, BIOFACT, Daejeon, Korea). The sequence analysis was performed at SolGent. The LCY-e1 and CRTISO primers were used as DNA markers for screening the genotype of *LCY-E* and *CRTISO*, respectively. The PCR products obtained using the DNA markers were separated by electrophoresis on 1% agarose gel and were visualized by staining with ethidium bromide.

## 4. Conclusions

Tomato fruits show various colors, such as red, pink, orange, yellow, brown, and purple. Among the diverse pigments affecting the fruit color, carotenoids are the primary determinants. Therefore, diverse color mutants often affect the carotenoid synthesis. Identification of genetic factors affecting the variation in fruit colors is time-consuming and labor intensive. Visual phenotypes underlying different genotypes are often indistinguishable from each other. Our study could distinguish similar fruit color variation by carotenoid profiling. Four distinct carotenoid profiles were investigated, and two candidate genes affecting fruit color and carotenoid variation were verified. Metabolite analysis using HPLC is an efficient tool for identifying the genes regulating the carotenoid biosynthesis in tomato. Metabolomics analysis in the field of genetics and breeding should be conducted for hypothesizing the mode of genetic action.

## Figures and Tables

**Table 1:** Identification of carotenoids in tomato fruits based on the retention time and absorption maxima over the observed wavelength.

Compound	RT (min)	λ (nm)						Observed Wavelength (nm)
		Observed			Reported			
Phytoene	16.9	(274) *	286	298	(276)	286	297	286
Phytofluene	17.6	330	350	366	331	348	367	348
	18.4							
Lutein	14.3	(418)	442	470	(421)	445	474	434
β-carotene	20.3	(422)	450	478	(425)	449	476	450
Prolycopene	20.6	414	438	(466)	414	436	463	450
δ-carotene	22.3	430	454	486	431	456	489	450
γ-carotene	23.5	438	466	494	437	462	494	450
*cis*-lycopene	25.1	446	470	502	446	472	503	471
*trans*-lycopene	26.4	446	470	502	444	470	502	471

* Parentheses indicate the values for the shoulder peaks observed in this study and as reported by Britton et al. [41].

**Table 2:** Primers of *LCY-E* and *CRTISO* used for sequence analysis and genotyping.

Marker Name	Primer Name	Sequence (5′–3′)	T_m_ (°C)
LCY-e1	LCY-e1-F	CATGTTTGAAAACAAGCCAATATTG	56.4
	LCY-e1-R	TGCTGGAGTTATTTCATCTTGAC	57.0
CRTISO	CRTISO-F	AGTCGAATCAATCTGAATTCACCT	57.5
	CRTISO-R	GGTCAAAACAAGAACTTCTCTGTT	57.5
Primers for *CRTISO* cloning	CRTISO-gDNA-1F	TCTTGGGTTTCCAGCAATTTAAAG	57.5
	CRTISO-gDNA-1R	AACTTCTAATTTACGTCCTACTGC	57.5
	CRTISO-gDNA-2F	TACTATTGTATATGGTCTGCAGTG	57.5
	CRTISO-gDNA-2R	AGAATTACCATGCTTGCATTGATC	57.5
	CRTISO-gDNA-3F	TTGCTGTCTTTTATAGATGCAGAG	57.5
	CRTISO-gDNA-3R	ATACTGGCAAAGACAACATTGAAG	57.5
	CRTISO-gDNA-4F	TCAGTTACATCCCTGAAGTTATTC	57.5
	CRTISO-gDNA-4R	GTAAACATAGATGGTGTAACTCCA	57.5
	CRTISO-gDNA-5F	CTGAAAGGATTATAAGCAGACTTG	57.5
	CRTISO-gDNA-5R	AATCTGTGATTTCTATACTGGAGG	57.5
